# In Vivo Evaluation of the Anti-Inflammatory Effect of* Pistacia lentiscus* Fruit Oil and Its Effects on Oxidative Stress

**DOI:** 10.1155/2016/6108203

**Published:** 2016-12-14

**Authors:** Sameh Ben Khedir, Masarra Mzid, Sana Bardaa, Dorsaf Moalla, Zouheir Sahnoun, Tarek Rebai

**Affiliations:** ^1^Research Unit of Anatomy-Histology and Embryology, Faculty of Medicine, Sfax, Tunisia; ^2^Research Unit of Pharmacology, Faculty of Medicine, Sfax, Tunisia

## Abstract

In order to find new topical anti-inflammatory agents, we had recourse to a medicinal plant. This work was designed to determine the topical anti-inflammatory effect of* Pistacia lentiscus* fruit oil (PLFO), using carrageenan-induced paw edema rat model, and to evaluate its effects on oxidative stress. The topical anti-inflammatory activity of PLFO was compared to Inflocine® and estimated by measuring the diameter of paw edema, for 5 hours at a 1-hour interval. After that the rats were scarified and the inflamed paw tissue was removed for the exploration of some parameters of oxidative stress and histopathology. PLFO showed a significant anti-inflammatory activity in comparison with the Inflocine. The percentages of edema inhibition were 70% and % 51.5% (*p* < 0.01), respectively, after five hours. The treatment with PLFO and Inflocine led to significant increases (*p* ≤ 0.05) in the activities of CAT, SOD, and GPX and significant decreases in the MDA level and AOPP activity in the paw tissue after Carr injection, in comparison with the Carr group. Therefore, our findings demonstrate that PLFO might accelerate the development of new drugs which could be used scientifically as a source for natural health products in the treatment of topical inflammation.

## 1. Introduction

Inflammation is a physiological defense mechanism resulting from an attack to the body to isolate and repair the tissue damage. It plays a protective role by participating in the process of innate defense of the body and manifests itself clinically by four cardinal signs such as redness, heat, pain, and edema.

In traditional medicine, plants have long been used as alternative treatments for a wide range of diseases, including inflammatory processes of diverse origins, and have provided symptomatic relief comparable to that obtained from allopathic medicines [1, 2].

Treating inflammation with the analgesic, nonsteroidal anti-inflammatory drugs (NSAIDs), and corticosteroids makes as us face a new era of people presenting with symptoms of analgesic abuse and its adverse effects like gastric discomfort, gastric erosion, hypersensitivity reactions, muscular weakness, precipitation of diabetes mellitus, and increased susceptibility to infection [3]. With the progress of more and more synthetic drugs which have adverse effects, it is time to consider indigenous herbal plants as possible remedies. This has sped up the global effort to collect those medicinal plants that have substantial beneficial effects with the least adverse effects.

Over the last 10 years, a significant body of evidence has emerged indicating that chemically diverse classes of naturally-occurring substances, derived from higher plants, are of potential interest for therapeutic interventions in several inflammatory diseases [1].

Most plant-derived secondary metabolites are known to affect directly or indirectly the following inflammatory molecules and/or mechanisms of inflammation.

The carrageenan (Carr) induced edema, as an inflammation model, is usually used to assess the contribution of natural products to resist the biochemical changes associated with acute inflammation. When Carr is injected, acute inflammation with edema appears, along with a production of free radicals as well as a release of inflammatory mediators [4]. Simultaneously, intracellular antioxidant mechanisms involve antioxidant enzymes, including superoxide dismutase (SOD), catalase (CAT), and glutathione peroxidase (GPx) to counteract this inflammatory stress in tissues. Actually, it has been shown that defective cellular antioxidant systems cause organisms to develop a series of inflammatory and cancer diseases [5]. Nevertheless, it appears that the various roles of enzymatic antioxidants help to protect organisms from excessive generation of oxidative stress in the inflammatory process [6]. This has incited studies focusing on the role of natural products in suppressing the production of oxidation by increasing enzymatic antioxidants in tissues [7].

*Pistacia lentiscus* L. is an evergreen plant of the Anacardiaceae family. It characterizes the Mediterranean region and it is commonly dispersed in Tunisia from the humid to the arid areas [8]. The fruits, galls, resin, and leaves of the* P. lentiscus* have a long tradition in folk medicine. They are used in eczema treatment, paralysis, diarrhea, throat infection, renal stones, jaundice, asthma, and stomach-ache, with astringent, anti-inflammatory, antipyretic, antibacterial, antiviral, pectoral, and stimulant properties [9–12]. Fruits of* P. lentiscus* give an edible oil which is rich in unsaturated fatty acids as oleic and linoleic [5]. In Tunisia, the* Pistacia lentiscus* fruit oil (PLFO) is used by the population as a traditional medicine in the treatment of scabies and rheumatism and in the manufacture of antidiarrhea pills [13]. The fruit oil is used internally for respiratory allergies, externally to treat sore throats, and locally applied for wounds and burns [14, 15].

The value of PLFO in health medicine may be justified based on the traditional uses of PLFO, regarding the particular features and other desirable physicochemical characteristics, and given the lack of scientific studies on its potential anti-inflammatory properties. The objective of this work mainly aimed to investigate the role of topical dermal administration of PLFO in alleviating inflammation of carrageenan-induced rat paw edema and its effects on oxidative stress. Also, attempts have been made to explore the possible mechanism of PLFO anti-inflammatory action in acute phases of inflammation.

## 2. Materials and Methods

### 2.1. Plant Material and Reagents

*P. lentiscus* fruits were collected from the region of El Kef (North West Tunisia). The fruits were authenticated by Dr. Hammadi Ben Salah and the voucher sample was deposited at The National Botanical Research Institute of Tunisia (INRAT). The PLFO was extracted from the freshly harvested fruits according to the traditional procedures used by the women living in the local area. However, Inflocine was served as a reference drug ointment. Inflocine is a NSAID and the most common anti-inflammatory drug, topically applied, used for self-medication and prescription, and available in Tunisian local pharmacies. This product is manufactured in Tunisia by IPS laboratory (medical authorization number 9273121). Niflumic acid 3% is reported to be the basic ingredient of Inflocine ointment. It is a nonsteroidal anti-inflammatory treatment recommended for pain and inflammation associated with musculoskeletal and joint disorders. The other ingredients of Inflocine include mono- and dipalmitostearate of polyethylene glycol, macrogol 400, liquid paraffin, saturated polyglycol glyceride, stearic acid, sodium methyl parahydroxybenzoate, sodium propyl parahydroxybenzoate, gamma perfume, and purified water.

Finally, Lambda carrageenan was purchased from Sigma Chemical Company (St. Louis, USA). It was used to induce paw edema. 1% w/v carrageenan was prepared by dissolving 1 gm of carrageenan in 100 mL of normal saline.

### 2.2. Methods

#### 2.2.1. Analysis of* Pistacia lentiscus* Fruit Oil by GC-MS

The chemical composition of PLFO was determined by GC analysis using a Perkin-Elmer Autosystem XL gas chromatograph equipped with a flame ionisation detector. The chemical separated components were identified in comparison with retention times by known standards. The chemical components were further identified by gas chromatography mass spectrometry (GC-MS).

#### 2.2.2. Animals

The research team conducted the experiment on Wistar rats with a body weight of around 180 g each. Animals were placed in Microlon boxes in a controlled environment (temperature 25°C ± 2°C), with a standard laboratory diet and water ad libitum. The experimental protocols were conducted in accordance with guidelines for the care and use of laboratory animals issued by the University of Sfax, Tunisia, and approved by the Committee of Animal Ethics. The animals were kept in separate cages to prevent the inflammatory areas from being licked or bitten by other animals.

#### 2.2.3. Model of Carrageenan-Induced Paw Edema

The anti-inflammatory activity was evaluated by the method of Carr-induced edema in the subplantar region of the right hind paw of the rats [16]. Edema was induced by an injection of 0.1 mL of 1% freshly prepared suspension of carrageenan.

This method was chosen over other methods for the discovery and evaluation of anti-inflammatory effect of PLFO, as it is the most basic method requiring minimal equipment, but much practice. The difference at the various time intervals gives some hints for the duration of the anti-inflammatory effect. In addition, inhibition of Carr-induced inflammation has been shown to be highly predictive of anti-inflammatory drug activity in human inflammatory diseases and the dose of NSAIDs in this model correlates with the effective dose that should be administered to patients.

The anti-inflammatory activity of the PLFO is compared to that of the nonsteroidal anti-inflammatory reference (Inflocine).

### 2.3. Anti-Inflammatory Activity

Wistar rats were divided into 4 groups, with 6 rats in each. The first group of rats had no inflammation and received no treatment. The second group was inflamed by carrageenan injection and did not undergo any treatment. The inflammation of the third group, used as reference, and that of the fourth group was treated by a topically dermal application with Inflocine (2 mg/paw) and PLFO (20 *μ*L/paw), respectively, 1 hour after the carrageenan injection. The doses of PLFO and Inflocine chosen during treatments were proportional in the size of the edema and covered the whole swelling. The PLFO and Inflocine were applied to the plantar surface of the hind paw by gentle rubbing 50 times with one's index finger.

In all treated groups, the size of the edema was measured before and after the inflammatory injection using a digital caliper [17]. Edema was expressed as the relative increase in paw volume induced by the inflammation injection (i.e., the edema was proportional to the volume difference between 0 hours and the other times, 1 hour, 2, 3, 4, and 5 hours, after carrageenan injection).

Percentile edema inhibition was calculated according to the following formula: Percentile inhibition = [1 − (*V*_*T*_/*V*_0_)] × 100. *V*_*T*_ represents the edema volume in the drug treated group. *V*_0_ represents the edema volume in the Carr group.

The degree of inflammation induced was evaluated according to the following formula: Percentile inflammation = (*P*_*T*_ − *P*_0_)/*P*_0_ × 100. *P*_*T*_ represents the volume of the right hind paw after Carr treatment. *P*_0_ represents the volume of the right hind paw before Carr treatment.

### 2.4. Exploration of Some Parameters of Oxidative Stress

#### 2.4.1. Preparation of Extracts

The oxidative stress parameters were determined in skin tissue edema. Homogenates were diluted (10%, w/v) in a phosphate buffer (pH 7.4) and centrifuged at 9000 rpm for 20 min. The resulting supernatants were used for oxidative stress assays.

#### 2.4.2. Protein Quantification

Skin protein contents were measured according to Lowry et al.'s (1951) [18] method using bovine serum albumin as a standard.

#### 2.4.3. Malondialdehyde (MDA) Levels

Lipid peroxidation was determined in tissue homogenates by the method of Draper and Hadley (1990) [19] following a reaction of thiobarbituric acid (TBA) with MDA formed owing to the peroxidation of lipids. After the incubation of tissue homogenates with TBA at 95°C during 10 min, the pink color produced by this reaction was determined spectrophotometrically at 532 nm. The malondialdehyde values were calculated using 1, 1, 3, 3-tetraethoxypropane as a standard and expressed as nmol of malondialdehyde per gram of tissue.

#### 2.4.4. Advanced Oxidation of Protein Product (AOPP) Levels

Advanced oxidation protein product levels were determined according to the method of Kayali et al. (2006) [20]. The absorbance of the reaction mixture was immediately recorded at 340 nm. The concentration of AOPP for each sample was calculated using the extinction coefficient of 261 cm^−1^ mM^−1^ and the results were expressed as *μ*moles per milligram of protein.

#### 2.4.5. Antioxidant Parameters (Vitamin C Levels)

Vitamin C determination was performed as described by Jacques-Silva et al. (2001) [21]. Protein in tissue homogenate was precipitated in 10 volumes of a cold 5% trichloroacetic acid solution. An aliquot of the sample (500 *μ*L) was mixed with a color reagent containing 4.5 mg/mL dinitrophenyl hydrazine and CuSO_4_ (0.075 mg/mL). The mixture was incubated at 85°C for 30 min, then 700 *μ*L of H_2_SO_4_, 65% (v/v), was added to the medium. The reaction absorbance of the cooled mixture was recorded at 540 nm. The data were expressed as *μ*mol ascorbic acid per gram of tissue.

#### 2.4.6. Antioxidant Enzyme Activities

*Superoxide Dismutase Activity (SOD)*. The superoxide dismutase (SOD) level was determined using Beauchamp and Fridovich's method (1971) [22]. The reaction mixture contained 50 mM of kidney homogenates in 0.1 M of potassium phosphate buffer (pH 7.4), 0.1 mM EDTA, 13 mM L-methionine, 2 mM riboflavin, and 75 mM Nitro Blue Tetrazolium (NBT). The developed blue color in the reaction was measured at 560 nm. Units of SOD activity were expressed as the amount of enzyme required to inhibit the reduction of NBT by 50% and the activity was expressed as units per mg of protein.

*Catalase Activity (CAT)*. CAT was assessed following the method of Aebi (1984) [23]. Briefly, 20 *μ*L of homogenate tissue was added to 880 *μ*L of H_2_O_2_ solution (containing 0.5 mol/L H_2_O_2_ and 0.1 mol/L, phosphate buffer, pH = 7.4). CAT activity was determined by monitoring the H_2_O_2_ decomposition which was measured spectrophotometrically by the decrease in absorbance at 290 nm. Enzyme activity was calculated using a molar extinction coefficient of 0.043/mM^−1^/cm^−1^ and expressed as *μ*mol H_2_O_2_ consumed/min/mg of protein (*μ*M/minute/mg protein).

*Glutathione Peroxidase Activity (GPx)*. Glutathione peroxidase (GPx) activity was determined according to Flohé and Günzler (1984) [24] method. One milliliter of the reaction mixture was prepared, containing 0.3 mL of phosphate buffer (0.1 M, pH 7.4), 0.2 mL of 2 mM glutathione (GSH), 0.1 mL of sodium azide (10 mM), 0.1 mL of H_2_O_2_ (1 mM), and 0.3 mL of tissue homogenates,. After incubation at 37°C for 15 min, their action was terminated by the addition of 0.5 mL 5% TCA. Tubes were centrifuged at 1500 g for 10 min and the supernatant was collected. 0.2 mL of phosphate buffer (0.1 M pH 7.4) and 0.7 mL composed of 5,50 dithiobis-(2-nitrobenzoic acid) and (DTNB, 0.4 mg/mL) were added to 0.1 mL of the supernatant. After mixing, absorbance was recorded at 420 nm and the enzyme activity was calculated as nmoles of GSH/min/mg protein.

### 2.5. Histopathology Examination

For histological examination, biopsies of paws were taken 5 h following the interplanetary injection of carrageenan. All tissue samples were fixed in 10% neutral buffered formalin solution, embedded in paraffin wax, cut into 5 *μ*m thick sections and stained with hematoxylin-eosin. All samples were photographed with a Canon EOS700 camera connected to an optical microscope.

### 2.6. Statistical Analysis

Statistical analyses were performed using SPSS version 17 (SPSS Inc., Chicago, Il, USA). Nonparametric tests, Kruskal-Wallis test, and Mann–Whitney test were used to compare edema in different groups. Raw data were shown with median IQR for each group. Differences were considered to be statistically significant at *p* < 0.05.

For antioxidant parameters, data are expressed as mean and standard error of the mean ± SEM). The one-way analysis of variance (ANOVA) and the Tukey post hoc test were performed on the data for intergroup comparisons. The nominal statistical significance level was set at 0.05.

## 3. Results

### 3.1. GC-MS Composition of* P. lentiscus* Fruit Oil

The PLFO was analyzed by GC-MS.  Table 1 presents the individual components, identified in the oil extract with their relative percentages. Overall, forty-one components were identified, representing 100% of the total oil.

The results revealed that the oil contained a complex mixture of several components, predominately hydrocarbons, monoterpenes, and sesquiterpenes. The major components identified were *α*-pinene (13.35%), *β*-phellandrene (10.45%), *α*-phellandrene (10.12%) sabinene (7.01%), germacrene-D (6.68%), 2-*β*-pinene (5.57%), *β*-caryophyllene (4.58%), and myrcene (4.33%).

### 3.2. Effects of PLFO on Carr-Induced Paw Edema

In this study, we used Carr-induced edema because this model is widely employed to screen the effects of anti-inflammatory drugs.

**Figure 1:**
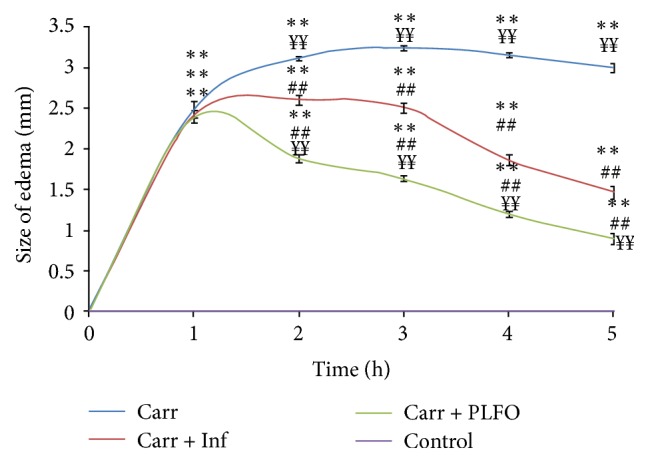
Effect of PLFO and Inflocine on hand paw edema induced by carrageenan. Results were obtained by dermal application of PLFO and Inflocine. Each value represents the mean ± SEM of results from six rats. ^*∗∗*^ *p* < 0.01 compared to control; ^##^ *p* < 0.01 compared to Carr; ^*¥¥*^ *p* < 0.01 compared to Carr + Inf.

**Figure 2:**
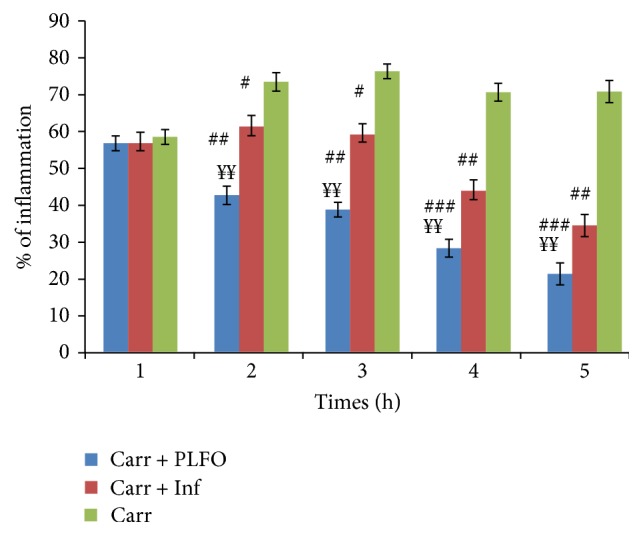
Percentage (%) of inflammation data in all groups. Values represent means ± SEM (*n* = 6) in each group. ^#^ *p* < 0.05, ^##^ *p* < 0.01, and ^###^ *p* < 0.001 compared to Carr; ^*¥¥*^ *p* < 0.01 compared to Carr + Inf.

Figure 1 shows that the induction of carrageenan in the rat hind paw started off the vascular phase of inflammation which generated an increase in the size of the edema for all groups. This injection generated intense inflammation which peaked after 3 hours. The experimental data showed that PLFO presented a significant (*p* < 0.01) inhibition of paw edema which was time dependent and more important than that of the standard Inflocine (reference drug) and the Carr group. The maximal inhibition percentage of the edema volume was observed after 5 h when compared to the control and reference groups. In the second hour, the inflammation and the inhibition (PI) percentages were, respectively, 42.44% and 42% in the PLFO group and 61.07% and 16.6% in the reference group. After five hours, the inflammation and the inhibition (PI) percentages were, respectively, 21.12% and 70% in the PLFO group and 34.27% and 51.5% in the reference group (Figure 2).

### 3.3. Changes in Oxidative Stress Parameters

Inflammation is a generating source of oxidative stress. It manifests by the excessive production of free radicals that attack biomolecules (phospholipids, proteins, and nucleic acids) and it can be demonstrated by measuring the oxidative damage to lipids and proteins.

#### 3.3.1. Lipid Peroxidation: MDA Assay

**Figure 3:**
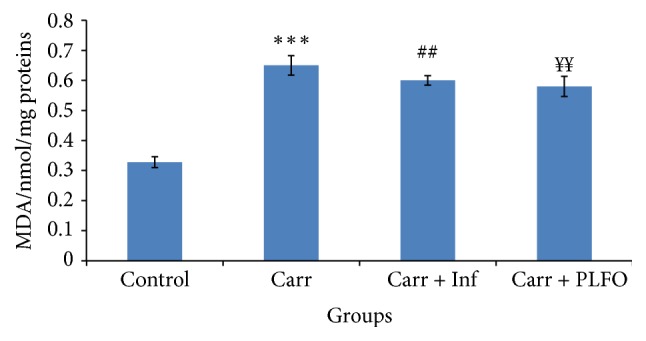
Levels of MDA in the paw of the control and treated rats with Carr, Inf, and PLFO. Values represent means ± SEM (*n* = 6) in each group. ^*∗∗∗*^ *p* < 0.001 compared to control; ^##^ *p* < 0.01 compared to Carr; ^*¥¥*^ *p* < 0.01 compared to Carr + Inf. MDA: malondialdehyde.

The assessment of dermal lipid peroxidation is illustrated in Figure 3. The figure reveals that the induction of inflammation with carrageenan induced a highly significant increase in the level of dermal MDA with percentages of 98.17% (*p* ≤ 0.001) in comparison with the control negative group (not inflamed and not treated). However, we noted that treatment with PLFO and Inflocine decreased significantly (*p* < 0.01) the level of lipid peroxidation by 18.18% and 12.08%, respectively, when compared to the Carr group.

In rats treated with PLFO (Carr + PLFO), a significant decrease was noted in the level of lipid peroxidation by (5.17%) in comparison with the reference group (Carr + Inf) (*p* ≤ 0.01).

#### 3.3.2. Protein Peroxidation: Determination of Advanced Oxidized Protein Products (AOPP)

**Figure 4:**
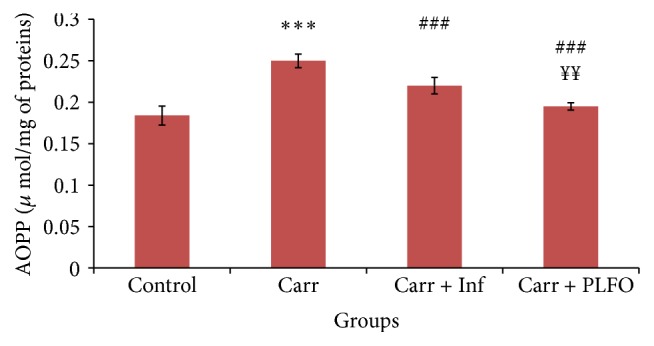
Levels of AOPP in the paw of the control and treated rats with Carr, Inf, and PLFO. Values represent means ± SEM (*n* = 6) in each group. ^*∗∗∗*^ *p* < 0.001 compared to control; ^###^ *p* < 0.001 compared to Carr; ^*¥¥*^ *p* < 0.01 compared to Carr + Inf. AOPP: advanced oxidation protein product.

Changes in primary, secondary, and tertiary structures of proteins are the basis of the formation of protein carbonyl derivatives. The dosage of AOPP in skin is illustrated in Figure 4. Our results demonstrated that the injection of carrageenan caused a highly significant increase in AOPP levels by 35.86% when compared with control group (*p* < 0.001). The rate of AOPP decreased significantly in rats treated with PLFO and Inflocine by 28.20% and 13.63%, respectively, when compared with inflamed untreated rats (Carr group).

Also, we noted a significant decrease in AOPP in the rats treated with PLFO by 12.82% (*p* < 0.001) when compared with the reference drug (Carr + Inf).

#### 3.3.3. Exploring the Antioxidant Status

The inflammatory reaction involves the overproduction of EOA, responsible for the generation of oxidative stress. Thus, the defense mechanisms against the production of free radicals involve antioxidant enzymes such as SOD, CAT, and GPx enzymes and vitamin C. To explore the antioxidant status, we measured the activity of these three enzymes and the rate of vitamin C in the dermal tissue.

#### 3.3.4. Activity of Superoxide Dismutase (SOD), Catalase (CAT), and Glutathione Peroxidase (GPx)

The subcutaneous injection of carrageenan led to a highly significant decrease in the activity of the dermal SOD in inflamed rats (Carr) by 57.51% compared with the control group (*p* ≤ 0.001). However, the application of PLFO and Inflocine increased significantly the activity of SOD compared with the untreated inflamed group (Carr) by 47.94% and 34.52%, respectively, (Table 3).

Additionally, the inflammation induced a significant decrease in catalase activity of Carr group (Table 3) by 37.73% compared to the noninflamed rats (control). In inflamed rats treated with PLFO (Carr + PLFO), the catalase activity showed a significant increase (*p* < 0.001) compared with inflamed untreated rats (Carr) (53.77%) and even that of the reference rats (Carr + Inf) (21.88%) (Table 3).

Indeed, the subcutaneous injection of carrageenan resulted in a significant decrease in the activity of GPx (Table 3) in the inflamed rats by 48.01% in comparison with control rats. The injection of PLFO increased significantly the activity of glutathione peroxidase (*p* < 0.001) by 79.97% compared with the Carr group and 20.71% when compared with Carr + Inf group.

#### 3.3.5. The Assay of Vitamin C

**Figure 5:**
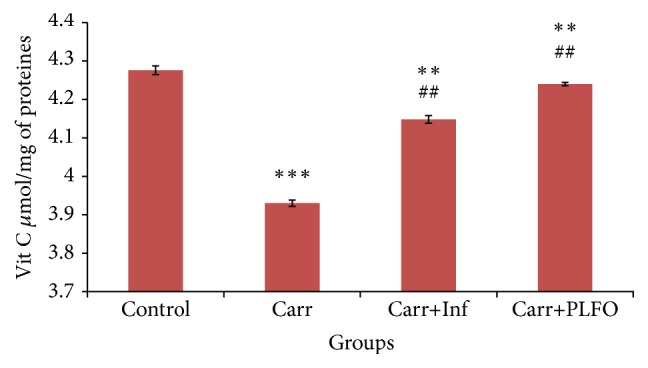
Nonenzymatic antioxidant levels (ascorbic acid (vitamin C)) in the paw of the control and treated rats with Carr, Inf, and PLFO. Values represent means ± SEM (*n* = 6) in each group. ^*∗∗*^ *p* < 0.01 and ^*∗∗∗*^ *p* < 0.001 compared to control; ^##^ *p* < 0.01 compared to Carr.

Vitamin C is considered as an antioxidant which traps free radicals. The dosage of the dermal vitamin C is illustrated in Figure 5.

Vitamin C decreased in the Carr group compared with the control group by 8.09%. However, it increased in the Car + PLFO and Carr + Inf groups by 7.88% and 5.34%, respectively, when compared with the Carr group.

### 3.4. Histopathology

**Figure 6:**
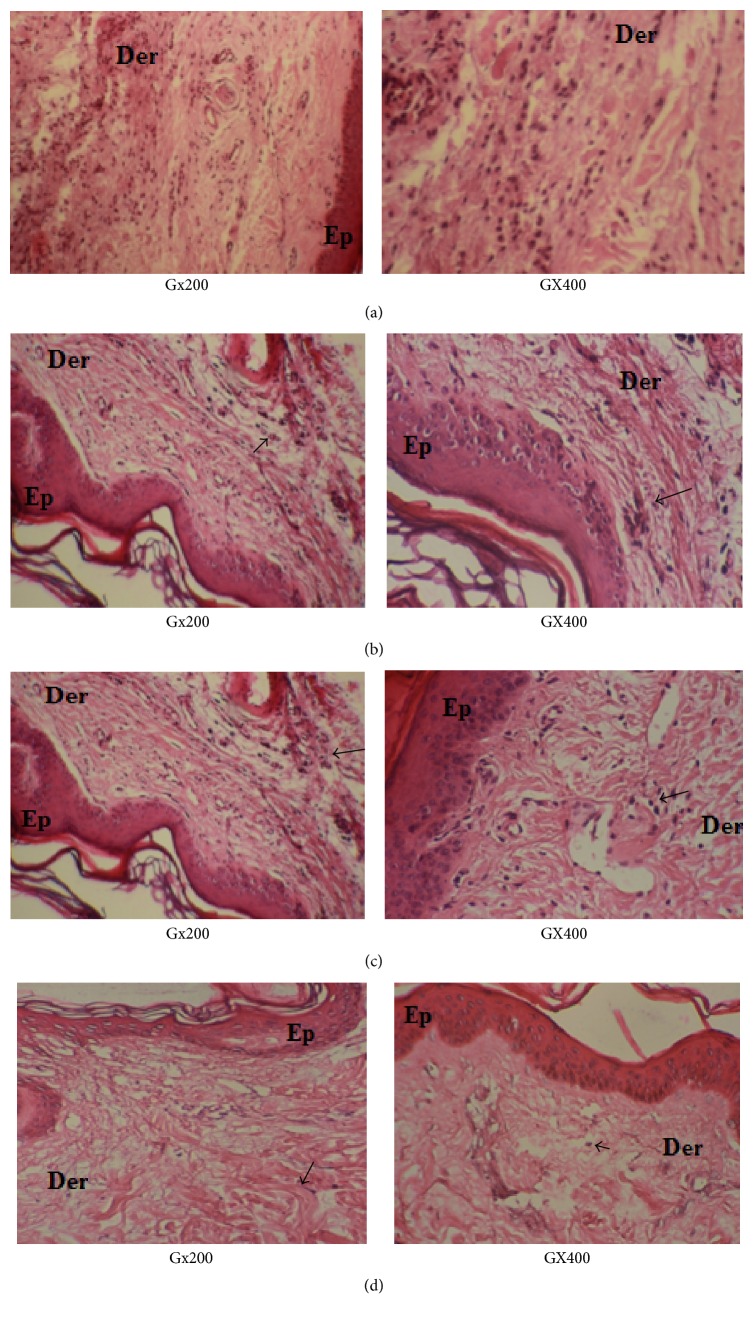
Representative photographs from the skin showing the protective effect of PLFO against carrageenan-induced inflammation in rats. Controls (a), rats treated with carrageenan (Carr) (b), the combination of Inflocine (Inf) + carrageenan (Carr) (c), and rats treated with the combination of Carr + PLFO (d). Ep: epidermis, Der: dermis, and →: lymphocytic infiltration.

A histopathological study was carried out in paw tissue 5 h after the induction of inflammation (Figure 6). A microscopic study of the paw biopsies of carrageenan model animals showed marked cellular diffuses infiltration in the connective tissue with acute edematasis in epidermis and dermis. The epidermis showed sponge-like appearance and bullae formed. There were also vasculitis and hyperaemia around the vessels in the dermis.

The paw biopsies of animals treated with PLFO and Inflocine showed a reduction in the inflammatory response (reduction of lymphocytic infiltration). In fact, inflammatory cells were reduced in number and imprisoned near the vascular areas as compared to the Carr-treated group (Figure 6).

## 4. Discussion

The inflammatory reactions can be generated by exogenous or endogenous aggression which is characterized by vascular and cellular events. These reactions successively induce the production of ROS.

Various external nonsteroidal anti-inflammatory drugs (NSAIDs) have been used as a topical external treatment, but the use of these drugs is sometimes restricted due to side effects. To avoid these adverse reactions many efforts have been made including the development of new compounds derived from medicinal plant. In developing countries, plants possessing an anti-inflammatory activity could establish an alternative to the anti-inflammatory therapeutics because of their best accessibility and their slightest toxicity in comparison with classic anti-inflammatory drugs [25].

In the present study the anti-inflammatory activity of PLFO was investigated by experimental carrageenan animal model and the concentrations of antioxidant enzymes were measured. The fact that PLFO possesses a significant anti-inflammatory activity in comparison with the Inflocine, a nonsteroidal anti-inflammatory drug, allows PLFO to be a potential and a novel topically applied anti-inflammatory therapeutic drug extracted from healthy oil.

The carrageenan test is highly sensitive to nonsteroidal anti-inflammatory drugs and has long been accepted as a useful model to determine the anti-inflammatory effects of natural products [26]. In addition, inhibition of Carr-induced inflammation has been shown to be highly predictive of anti-inflammatory drug activity in human inflammatory diseases and the dose of NSAIDs in this model correlates with the effective dose that should be administered to patients [27].

Indeed, the carrageenan injection produced a biphasic event in the inflammation mechanism. In the vascular phase (first phase) (1 h–3 h), proinflammatory cytokines like TNF-*α*, IL-6, and IL-1*β*, histamine, and serotonin are the mediators of inflammation that were involved during the first hour. Bradykinin is freed during the period between 1.30 and 3 hours while prostaglandins are implied in the cellular phase (second phase) (3–5 hours) [28]. These mediators are responsible for redness, edema, and secretions.

In our study, the induction of carrageenan in the rat hind paw started off the vascular phase of inflammation which was characterized by temporary vasoconstriction and vasodilatation that generated an increase in the size of the edema for all groups. This injection generated intense inflammation, which peaked after 3 hours. These results correlate with previous studies showing that 3 hours after the carrageenan injection is the moment when its maximum effect is manifested and the moment when the anti-inflammatory activity of the test product is best observed [29]. The experimental data showed that PLFO presented a significant (*p* < 0.01) paw edema inhibition which was more important than that of the standard Inflocine (reference drug) and Carr group. The inhibition of the paw edema by PLFO was time dependent. This suggested that PLFO has an antagonistic action to all mediators of inflammation implicated in the vascular and cellular phases. In addition, the strong inhibition of the edema was observed after 5 hours; this suggests that the maximum inhibitory action of PLFO can be exerted by the cyclooxygenase that is responsible for prostaglandin synthesis.

Similar to medicinal plants used in folk medicine, the anti-inflammatory activity of PLFO could be due to its phytochemical compounds such as fatty acids, monoterpenes, and sesquiterpenes.

It has been described that polyunsaturated fatty acids function as mediators and regulators of inflammation. Dhifi et al. (2013) showed that the total unsaturated fatty acid of PLFO is 73.58%. The major components are oleic (51.06%), linoleic (20.71%), and palmitic acids (23.52%) [30]. Unsaturated fatty acids are able to influence the biochemical properties of the membrane such as fluidity and permeability [31]. It has been reported that gradual consumption of polyunsaturated fatty acids in cell membranes results in a partial replacement of the arachidonic acid by eicosapentaenoic and docosahexaenoic acids. This leads to reduced production of arachidonic acid-derived mediators and, therefore, is a potentially beneficial anti-inflammatory effect of unsaturated fatty acids. Moreover, unsaturated fatty acids have a number of other effects that might occur downstream of altered eicosanoid production or might be independent of this activity. For example, they result in suppressed production of proinflammatory cytokines and can modulate adhesion molecule expression [32]. In addition, the major fatty acids in PLFO are able to inhibit the activity of COX-1 and COX-2 [1].

Monoterpene and sesquiterpene compounds can exhibit marked anti-inflammatory effects in vascular and/or cellular phase. Indeed, monoterpenes identified by GC-MS in PLFO such as humulene, caryophyllene, *β*-pinene, and *α*-pinene can exhibit marked anti-inflammatory effects in the cellular phase. These monoterpenes act as cyclooxygenase [33].

It has been demonstrated that humulene and caryophyllene exhibit oral and topical anti-inflammatory properties in different inflammatory models by reducing inflammatory cytokine levels and proinflammatory protein expression via inhibition of the nuclear factor kB (NF-kB) pathway [34–36]. The *β*-pinene (5.75%) and *α*-pinene (13.35%) that are also identified in PLFO inhibit the synthesis of nitric oxide (NO), suggesting an antioxidant effect [37], and recent reports showed that *β*-pinene exerts an antispasmodic effect on the rat ileum, provoking antinociceptive actions [38] and exhibited a high antiedematous effect. The *α*-pinene isolated from* Pistacia vera* exudates had dose dependent anti-inflammatory effect on carrageenan-induced hind paw edema animal model [39].

Knowing that sesquiterpenes have excellent anti-inflammatory activities [40, 41], the anti-inflammatory activity of PLFO could be partly explained by the presence of some sesquiterpenes, especially cadinene, amorphene, caryophyllene, and muurolene (Table 2). For example, *β*-caryophyllene reduces the expression of TNF-*α*, IL-1*β*, interferon-*γ*, and keratinocyte-derived chemokine [42].

In addition, several secondary metabolites identified in the fruit of* P. lentiscus,* passing in fixed oil and accounting for 38.8% of the weight of fruit, can exhibit anti-inflammatory activity. Gallic acid, a polyphenol identified in the fruit of* P. lentiscus* [43], and its derivatives are responsible for inhibiting the activation of p38 and the binding inhibition NF-kappa B essential for the expression of proinflammatory cytokines such as histamine, TNF-*α*, and IL-6 [44]. Indeed, It has been reported that gallic acid in turn inhibits the migration of leukocytes by inhibiting molecules adhesion of VCAM-1, ICAM-1, and E-selectin in vascular endothelial cells. This inhibition is due to inhibition of IL-1, TNF-*α*, and NF-kB [45].

According Longo and colleagues (2007), the fruits of* P. lentiscus* contain 5.4 mg/mL of anthocyanins [46]. Anthocyanins block the migration of leukocytes to the inflammatory site by inhibiting adhesion of ICAM-1 and VCAM-1 molecules, with regulation by TNF-*α*. Tsuda et al. (2002) [47] report that the administration of cyanidin 3-O-beta-glucoside, which represents 70% of anthocyanins in fruits of PLFO, inhibits inflammation induced by zymosan and reduces the increase in concentrations of NO, TNF-*α*, IL-1*β*, and CINC-1 (cytokine-induced neutrophil chemoattractant). Finally, flavonoids identified in fruit of* P. lentiscus* [48] can inhibit leukocyte migration by blocking their adhesion to the vascular wall [49, 50]. This effect is due to inhibition of the synthesis of IL-1 and TNF-*α*, a major inducer molecule expression adhesive on the vascular wall [51].

Taking these data together, we can conclude that PLFO most likely decreased the paw edema by acting at both phases of the carrageenan-induced inflammation. The PLFO exerts its anti-inflammatory effect by reducing the production of inflammatory mediators involved in the conduct of stages of the acute inflammatory response induced by the *λ*-carrageenan and by inhibiting the leukocyte recruitment to the inflammatory site by exerting antichemoattractant effort on these effects and blocking the synthesis of prostaglandins by inhibition of cyclooxygenase.

The anti-inflammatory effect of PLFO was also confirmed by histological assessment. Indeed, the oil considerably reduced the morphological injury and neutrophil infiltration in a carrageenan model that induced local inflammation.

Inflocine applies its maximum inhibition (58, 12%) at 5 h, and shows effect only after 3 hours. It is related to its mechanism of action which acts by blocking the synthesis of prostaglandins by inhibiting cyclooxygenase, which converts arachidonic acid to cyclic endoperoxides, precursors of prostaglandins. The inhibition of prostaglandin synthesis accounts for their analgesic, antipyretic, and platelet-inhibitory actions [52].

On the other hand, the local inflammatory response is manifested by the generation of active oxygen species (ROS) involved in the genesis of oxidative stress [53]. Defense systems act against the oxidative stress condition while involving a defense mechanism based on the production of antioxidant enzymes such as superoxide dismutase (SOD), catalase (CAT), and glutathione peroxidase (GPx) [54].

According to our results, the induction of inflammation by carrageenan has resulted in a significant amplification (*p* ≤ 0.05) of MDA rates at the inflamed tissue. Indeed, the increase in MDA rates reflects the excessive production of the free radicals which induces an increase in biomarkers of oxidative stress. In contrast, after 5 hours, a significant decrease (*p* ≤ 0.05) in the rate of tissue lipid peroxidation was observed in treated rats with the PLFO and the reference product (Inflocine) which was coupled by to the reduction of edema size.

We found that a carrageenan injection caused a significant decrease in activity of SOD, CAT, and GPx at the inflamed tissue (Carr group) of 57.51%, 37.73%, and 48.01%, respectively, compared with the control group (*p* ≤ 0.05). This reduction in activity may be explained by high use of the produced enzymes that act as scavengers of free radicals generated during the inflammatory process and by the fact that their expression is not yet performed for five hours after induction of inflammation. Contrariwise, The topical application of PLFO and reference product (Inflocine) in inflammatory site increased the activities of SOD, CAT, and GPx tissue of 79.97%, 53.77%, and 47.94% and 44.08%, 21.85%, and 34.52%, respectively, compared with the inflamed untreated group (Carr group). It should be noted that the PLFO effect is more important than Inflocine effect (*p* ≥ 0.05) at tissue level.

These results suggest that the topical application of PLFO was involved in cellular protection not only in a straightforward manner as a source of antioxidant molecules but also indirectly as a stimulator of the activity and the expression of antioxidant enzymes. Thus, the antioxidant ability of various compounds in PLFO has also been the subject of our considerable interest.

First, the monoterpenes act as scavengers of free radicals generated during the inflammatory process [55]. Second, the antioxidant effect of PLFO is most likely due to its secondary metabolites which presented a high antioxidant capacity such as polyphenols, tocopherols, and sterols. The phenolic compounds present in the PLFO have the ability to trap the radical species and reactive oxygen species [56]. In fact, the phenolic compounds, which are able to interact with biological systems and act as bioactive molecules in particular, are important inhibitors of lipid peroxidation [57].

In PLFO, *α*-tocopherol scavenges free radicals and inhibits lipid peroxidation mechanism, which results in the formation of prostaglandins, physiological mediators of inflammation. Many pharmacological studies have shown that topical application of vitamin E decreased erythema and edema.

Polyphenols and tocopherols mainly act as chain breakers by donating radical hydrogen to alkylperoxyl radicals formed during the propagation step of lipid oxidation. The tocopherols constitute the lipophilic antioxidant group and are noted for their effective inhibition of lipid oxidation in all vegetable oils [58].

Finally, in PLFO, the total content of sterols was 4.17 g/kg, with *β*-sitosterol as the predominant sterol accounting for more than 55% [30]. Several studies suggest that phytosterols like stigmasterol are responsible, in part, for the prevention of disease development due to reactive oxygen species [59]. Moreover, Yoshida and Niki (2003) [60] have shown the antioxidant effect of phytosterols such as *β*-sitosterol, stigmasterol, and campesterol, against lipid peroxidation.

To synthesize, the anti-inflammatory and antioxidant activity of PLFO were carried by a synergistic effect between the major and minor constituents. On the one hand, The anti-inflammatory effect of PLFO is produced by reduction of the production of inflammatory mediators involved in the conduct of stages of the acute inflammatory response induced by the *λ*-carrageenan, as well as inhibition of leukocyte recruitment by exerting antichemoattractants effects and blocking the synthesis of prostaglandins by inhibition of cyclooxygenase on the latter. On the other hand, the antioxidant activity of PLFO was involved in cellular protection not only in a straightforward manner as a source of antioxidant molecules but also indirectly as a stimulator of the activity and the expression of antioxidant enzymes.

## 5. Conclusion

To the best of our knowledge, this is an unprecedented report of in vivo anti-inflammatory activity of PLFO and its effect on oxidative stress. The results indicate that the best anti-inflammatory activity was obtained after dermal application of PLFO on inflamed skin.

The interesting anti-inflammatory activity and its antioxidant effect recorded may support at least in part the traditional use of PLFO in the Mediterranean folk medicine. Considering the chemical composition of PLFO, it has been proved that these activities are not always related to its major constituents. Since PLFO is a complex natural mixture of fatty acids, monoterpenes, sesquiterpenes, polyphenol, flavonoids, tocopherols, and sterols metabolites, its biological activities (anti-inflammatory and antioxidant) may be related to synergistic interaction of both the major and minor components within the oil. PLFO exerts its anti-inflammatory effect by two mechanisms. First, there is a reduction of inflammatory mediator production involved in the conduct of stages of the acute inflammatory response, induced by the *λ*-carrageenan. Second, there is an inhibition of leukocyte recruitment to the inflammatory site. This occurs by exerting antichemoattractant effects and blocking the synthesis of prostaglandins by the inhibition of cyclooxygenase.

The effects of PLFO inflammation were also confirmed by histological assessment. The oil considerably reduced the morphological injury and neutrophil infiltration in a carrageenan model that induced local inflammation.

In addition, the antioxidant activity of PLFO was involved in cellular protection, not only in a straightforward manner as a source of antioxidant molecules but also indirectly as a stimulator of the activity and the expression of antioxidant enzymes.

According to its chemical compositions and biological activities, PLFO could contribute to a better utilization of this natural material in anti-inflammatory dermal treatment.

## Figures and Tables

**Table 1:** Composition of *P. lentiscus* fruit oil obtained by GC-MS.

Number	Rt (min)	Compound	(%)
1	6.264	Tricyclene	0.92
2	6.386	Alpha phellandrene	10.12
3	6.592	Alpha-pinene	13.35
4	6.878	Camphene	2.88
5	7.461	Sabinene	7.01
6	7.535	2-Beta-pinene	5.57
7	7.821	Myrcene	4.33
8	8.393	Alpha-terpinene	1.59
9	8.599	p-Cymene	2.16
10	8.726	Beta-phellandrene	10.45
11	9.086	Trans-beta-ocimene	0.78
12	9.240	Butanoic acid	0.48
13	9.346	Gamma-terpinene	2.33
14	9.997	Terpinolene	1.7
15	10.039	2-Nonanone	0.64
16	10.807	Chrysanthenone	0.24
17	11.252	Camphor	0.55
18	11.978	1-4-Terpineol	1.24
19	12.269	Alpha-terpineol	0.20
20	13.354	Hexanoic acid	0.18
24	15.404	Alpha-cubebene	0.27
25	15.938	Alpha-copaene	1.10
26	16.224	Beta-elemene	1.01
27	16.807	Beta-caryophyllene	4.58
28	17.331	Alpha-copaene	0.38
29	17.416	Alpha-humulene	1.27
30	17.538	Neoalloocimene	0.61
31	17.739	Epi-bicyclosesquiphellendrene	0.36
32	17.808	Gamma-muurolene	1.86
33	17.935	Germacrene-D	6.86
34	18.205	Alpha-muurolene	1.87
35	18.475	Alpha-amorphene	0.76
36	18.613	Delta-cadinene	3.55
37	18.782	Naphthalene,1,2,3,4,4a,7-hexahydro-1-6-dimethyl-4-(1-methylethyl)	0.2
38	19.703	Nopinone	0.48
40	23.484	Neophytadiene	0.21
41	32.936	1,2-Benzenedicarboxylic acid,bis (2-ethylhexyl)	3.87

**Table 2:** Statistical study of paw edema: Kruskal-Wallis and Mann–Whitney tests.

	Control	Carr	Carr + Inf	Carr + PLFO	*p value* (Kruskal Wallis)
0 h	0	0	0	0	1
1 h	0	2.51 (2.36–2.62)^*∗∗*^	2.37 (2.33–2.50)^*∗∗*^	2.44 (2.31–2.45)^*∗∗*^	<0.0001
2 h	0	3.13 (3.09–3.15)^*¥¥*,*∗∗*^	2.60 (2.60–2.53)^##,*∗∗*^	1.80 (1.75–1.88)^*¥¥*,##,*∗∗*^	<0.0001
3 h	0	3.25 (3.19–3.28)^*¥¥*,*∗∗*^	2.50 (2.46–2.60)^##,*∗∗*^	1.64 (1.59–1.68)^*¥¥*,##,*∗∗*^	<0.0001
4 h	0	3.14 (3.12–3.19)^*¥¥*,*∗∗*^	1.88 (1.75–1.92)^##,*∗∗*^	1.19 (1.17–1.25)^*¥¥*,##,*∗∗*^	<0.0001
5 h	0	3.02 (2.90–3.05)^*¥¥*,*∗∗*^	1.46 (1.38–1.58)^##,*∗∗*^	0.89 (0.82–0.99)^*¥¥*,##,*∗∗*^	<0.0001

Nonparametric tests: Kruskal-Wallis and Mann–Whitney tests. Raw data were shown with median IQR. ^*∗∗*^ *p* < 0.001 compared to Control, ^##^ *p* < 0.001 compared to Carr, and ^*¥¥*^ *p* < 0.001 compared to Carr + Inf.

**Table 3:** Antioxidant enzymes activity of GPx, SOD, and CAT in the paw tissue of the control and treated rats with Carr, Carr + Inf, and Carr + PLFO.

Groups	Control	Carr	Carr + PLFO	Carr + Inf
SOD (U/mg protein)	4,166 ± 0,0213	1,825 ± 0,0529^*∗∗∗*^	2,700 ± 0,00818 ^*∗∗∗*,###,*¥*^	2,4550 ± 0,005^*∗∗∗*,###^
CAT (*µ*mol H_2_O_2_/*µ*g of protein)	4,850 ± 0,02	3,02750 ± 0,02^*∗∗∗*^	4,644 ± 0,172 ^*∗*,### ,*¥¥¥*^	3,68125 ± 0,04^*∗∗∗*,###^
GPx (nmol GSH oxidized min/mg protein)	14,714 ± 0,03	7,647 ± 0,039^*∗∗∗*^	13,750 ± 0,040^*∗∗∗*,### ,*¥¥¥*^	11,390 ± 0,07^*∗∗∗*,###^

Values represent means ± SEM (*n* = 6) in each group. ^*∗*^ *p* < 0.05 and ^*∗∗∗*^ *p* < 0.001, compared to Control; ^###^ *p* < 0.001, compared to Carr; ^*¥*^ *p* < 0.005 and ^*¥¥¥*^ *p* < 0.001, compared to Carr + Inf; GPx: glutathione peroxidase, SOD: superoxide dismutase, and CAT: catalase.

## Abbreviations

AOPP: Advanced oxidation protein product
Carr: Carrageenan
Carr group: Group of rats inflamed by carrageenan and untreated
Carr + Inf: Group of rats inflamed by carrageenan and treated with Inflocine
Carr + PLFO: Group of rats inflamed by carrageenan and treated with* Pistacia lentiscus* oil
CAT: Catalase
COX-1: Cyclooxygenase 1
COX-2: Cyclooxygenase 2
GC-MS: Gas chromatography mass spectrometry
GPx: Glutathione peroxidase
DTNB: Dithiobis-(2-nitrobenzoic acid)
ICAM-1: Intercellular adhesion molecule 1
IL-1*β*: Interleukin 1 beta
INRAT: The National Botanical Research Institute of Tunisia
Inf: Inflocine
NBT: Nitro Blue Tetrazolium
NO: Nitric oxide
NSAIDs: nonsteroidal anti-inflammatory drugs
NF-kappa B: Nuclear factor
PI: Percentage of inhibition
PLFO: * Pistacia lentiscus* fruit oil
TBA: Thiobarbituric acid
TNF-*α*: Tumor necrosis factor
NF-*κ*B: Kappa-light-chain-enhancer of activated B cells
SOD: Superoxide dismutase
VCAM-1: Vascular cell adhesion molecule 1.
